# Pozzolanic Activity Assessment of LUSI (LUmpur SIdoarjo) Mud in Semi High Volume Pozzolanic Mortar

**DOI:** 10.3390/ma5091654

**Published:** 2012-09-17

**Authors:** Djwantoro Hardjito, Antoni, Gunadi M. Wibowo, Danny Christianto

**Affiliations:** Civil Engineering Department, Petra Christian University, Jalan Siwalankerto 121-131, Surabaya 60236, Indonesia; E-Mails: antoni@petra.ac.id (A.); koean_g@yahoo.com (G.M.W.); blind_angelo@live.com (D.C.)

**Keywords:** LUSI mud, mud volcano, pozzolanic material, strength activity index, water demand

## Abstract

LUSI mud obtained from the mud volcano in Sidoarjo, Indonesia, is a viable aluminosilicate material to be utilized as pozzolanic material. LUSI is an abbreviation of the local name of the mud, *i.e.*, Lumpur Sidoarjo, meaning Sidoarjo mud. This paper reports the results of an investigation to assess the pozzolanic activity of LUSI mud, especially in semi high volume pozzolanic mortar. In this case, the amount of mud incorporated is between 30% to 40% of total cementitious material, by mass. The content of SiO_2_ in the mud is about 30%, whilst the total content of SiO_2_, Fe_2_O_3_ and Al_2_O_3_ is more than 70%. Particle size and degree of partial cement replacement by treated LUSI mud affect the compressive strength, the strength activity index (SAI), the rate of pozzolanic activity development, and the workability of mortar incorporating LUSI mud. Manufacturing semi high volume LUSI mud mortar, up to at least 40% cement replacement, is a possibility, especially with a smaller particle size of LUSI mud, less than 63 μm. The use of a larger percentage of cement replacement by LUSI mud does not show any adverse effect on the water demand, as the flow of the fresh mortar increased with the increase of percentage of LUSI mud usage.

## 1. Introduction

This year is marked as the sixth year of a mud volcano eruption, nicknamed LUSI (a short form of LUmpur SIdoarjo or Sidoarjo mud), in Sidoarjo, Indonesia, which was started in May 2006. Since then, the mud has covered an area not less than 640 hectares, immersed productive land, industrial area and many villages; including several schools. The height of the cover dam is currently about 12 meters. It has discharged approximately 18 × 10^4^ m^3^/day of mud in its peak in 2007, and it continues until present, although the volume is decreasing to about 10^4^ m^3^/day. Several attempts have been carried out to end the eruption, however to date none were successful. It has been predicted that there is a 50% chance the eruption will last less than 41 years and a 33% chance that it will last more than 84 years [1].

A number of studies have been performed to utilize the LUSI mud for construction materials. It was found that the mud has a chemical composition similar to pozzolan, with SiO_2_ content ~55%, Al_2_O_3_ ~20% and Fe_2_O_3_ ~10% [2,3]. However, its crystalline properties causes the mud to be unreactive and thus it needs pre-treatment before usage [2,4], either as pozzolanic material or as source material for geopolymer [5]. Calcination or sintering is a common practice to increase the pozzolanic activity of pozzolanic material [6]. Nuruddin et al [3] applied sintering at 600 °C for a duration of one hour to convert its crystalline microstructure to an amorphous one. They concluded that the mud could be used as pozzolanic material to partially replace the use of cement in making mortar, with the optimum amount of 10%. The use of treated mud of 15% resulted in an approximately similar compressive strength of mortar without any cement replacement on the 28th day of age.

The reported results are promising. However, the amount of mud that can be incorporated into the making of mortar was considered low. Attempts have to be made to maximize the use of the mud. This study focuses on the possible use of semi high volume LUSI mud as a partial replacement for cement in making mortar. This paper reports the findings of the influence of particle size of the mud on the strength and strength activity index (SAI) of mortar. Furthermore, the effect of using higher amounts of cement replacement, up to 40% by LUSI mud, on the rate of the strength development is discussed. The results are correlated to examine the possibility of incorporating semi high volume LUSI mud as partial replacement for cement in making mortar or concrete.

## 2. Results and Discussion

### 2.1. Microstructural Analyses

**Figure 1:**
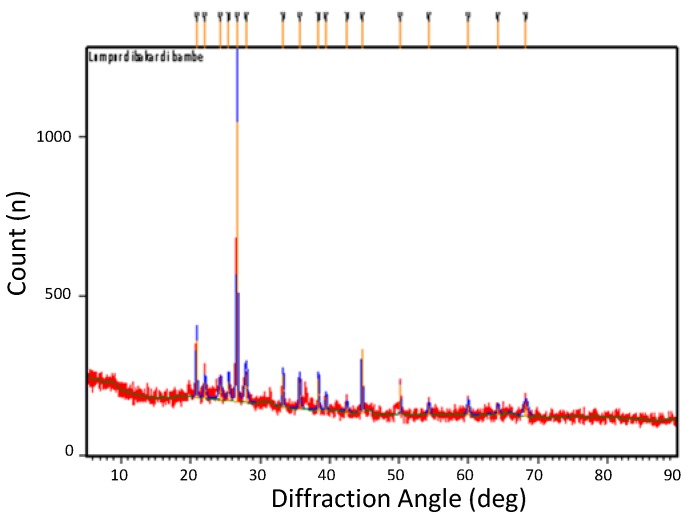
XRD result of sintered LUSI mud.

The chemical compositions of sintered mud taken from five different locations of the mud volcano are found to be similar to each other. The chemical composition of the unsintered mud is also very similar of those of sintered ones. Table 1 shows the typical chemical composition of sintered mud as measured by XRF (X-Ray Fluorescence), whilst Figure 1 describes the amorphous properties of mud, as also revealed in previous report [4].

**Table 1:** Chemical composition of sintered LUSI mud as measured by XRF (% by mass).

Chemical Composition of Sintered LUSI Mud	% by mass
CaO	7.36
SiO_2_	31
Al_2_O_3_	5.6
Fe_2_O_3_	42.79
K_2_O	4.03
SrO	0.4
SO_3_	1
MnO	0.65
ZnO	1.24
CuO	0.39
P_2_O_5_	2
PbO	0.16
TiO_2_	2.73
ZrO_2_	0.27
V_2_O_5_	0.13

It is found that the chemical composition of the LUSI mud is significantly different to those that have been reported earlier [2,3]. The SiO_2_ content is lower, whilst the Fe_2_O_3_ content is much more dominant. The results reveal that the chemical composition of the LUSI mud taken from different vertical layers may differ significantly, whereas those taken from various positions on the same horizontal layer show very similar properties. However, the total content of SiO_2_, Fe_2_O_3_ and Al_2_O_3_ is more than 70%, and thus LUSI mud shows potential to be used as pozzolanic material.

### 2.2. Compressive Strength and Strength Activity Index (SAI) as Function of Particle Size of LUSI Mud

In this series of experiments, four mixes were prepared utilizing the same amount of LUSI mud, *i.e.*, 20%, as partial replacement of cement. Water to cementitious ratio was kept constant at 0.5, whilst the sand to cementitious materials ratio was 2.5, all by mass. Three different particle sizes of LUSI mud were chosen for the investigation, *i.e.*, 150–300 μm, 63–150 μm and <63 μm.

SAI is a measure of the pozzolanic activity of pozzolanic material, as the strength relative to the control, in %. Specimen LM00_1 with no mud is taken as the control specimen. To be classified as a pozzolan, the SAI must be higher than 75% at 7-day and 28-day of age [7].

Table 2 shows, as expected, the smaller the particle size of the LUSI mud incorporated as pozzolanic materials for making mortar, the higher the compressive strength. Reducing the particle size of LUSI mud increases its reactivity as pozzolanic materials, as shown by the increase of its compressive strength and SAI. Incorporation of 20% mud with a particle size of 150 μm or less shows satisfactory SAIs for both 7-day and 28-day, which are higher than 75%. SAI for all specimens containing mud increased significantly with age.

**Table 2:** Compressive strength and Strength Activity Index (SAI) as a function of particle size.

Specimen	Particle size of LUSI mud	7-day		14-day		28-day		56-day	
		fc‘	7D SAI	fc‘	14D SAI	fc‘	28D SAI	fc‘	56D SAI
		(MPa)	(%)	(MPa)	(%)	(MPa)	(%)	(MPa)	(%)
**LM00_1**	No mud	37	100	48	100	50	100	51	100
**LM20S63**	<63μm	31	85	38	80	42	84	45	88
**LM20S150**	63–150μm	27	75	36	76	39	79	43	84
**LM20S300**	150–300μm	26	71	33	70	38	76	43	84

### 2.3. Mud Content, Compressive Strength and Strength Development

Three mixes with various content of semi high volume LUSI mud were prepared. The particle size of the LUSI mud was between 63 μm to 150 μm. In this series, water to cementitious ratio by mass was kept constant at 0.4 (except for LM00_1 where w/c was kept at 0.5), lower than that of the previous series, in order to achieve higher compressive strength. To improve its workability, superplasticizer was added at the amount of 1% of the cementitious material, by mass.

Table 3 shows the compressive strength of mortar at the age of 7, 14, 28 and 56^-^days with different mud content of 30%, 35% and 40% respectively, and its strength development relative to its initial 7-day strength. With the increase in the LUSI mud content, the relative increase in strength is larger. For mortar with no mud LM00_1, the 28-day strength increased 37% compared to its initial 7-day one, whilst its 56-day strength does not differ much from its 28-day one. On the other hand, mortar with 30% mud shows higher increment of 45% at 28-day and it continues to grow in its strength to 79% increment at 56-day. The largest increment is achieved by mortar with 40% mud, with its 56-day strength nearly double of its 7-day strength. This shows that the rate of pozzolanic reaction of LUSI mud slower than the hydration rate of OPC in early age, as shown by lower strength of mortar with mud, however it increases significantly with the increase in mud content and with age. It is expected that at later ages, the strength of mortar with mud content will continue to increase at higher rate, and thus will increase its SAI. In this regard, correlating the results with those presented in Table 2, one can expect that utilizing semi high volume LUSI mud up to at least 40% as cement replacement is a possibility, especially with a smaller particle size of LUSI mud, less than 63 μm.

**Table 3:** Compressive strength development as a function of mud content.

Specimen	Mud content	fc‘ (7-day)		fc‘ (14-day)		fc‘ (28-day)		fc‘ (56-day)	
		(MPa)	%	(MPa)	%	(MPa)	%	(MPa)	%
**LM00_1**	0%	37	100	48	129	50	137	51	139
**LM30S150**	30%	28	100	35	125	40	145	50	179
**LM35S150**	35%	23	100	29	129	37	163	44	190
**LM40S150/**	40%	22	100	28	127	37	170	42	190

### 2.4. Workability

Workability of the fresh mixes from both series of the experiments has been measured in term of its flow using the Flow Table apparatus. Flow was measured as the resulting increase in the base diameter of mortar, expressed as percentage of the original or initial diameter [8]. Table 4 shows the flow of fresh mixes. Indication of segregation was not found.

With the increase in the amount of mud, at least up to 40%, utilized as partial substitute for cement, the flow is also increasing. These results reveal important characteristics of sintered LUSI mud. The higher the amount of the mud, the bigger is the flow of fresh mortar. This means that the incorporation of LUSI mud does not have any adverse effect on water demand of the fresh mixes. On the contrary, it helps in improving the workability of the fresh-state mortar. This is similar to the case of utilizing fly ash in mortar or concrete, whereby its spherical shape helps in improving the workability of the fresh mix. In the case of LUSI mud, the mechanism is still not clear yet, and thus it needs further work to clarify. It might be due to particles interactions or the presence of electric charge in the mud particles to prevent flocculation to occur and thus reduces the water demand.

**Table 4:** The flow of fresh mixes.

Specimen	Particle size (μm)	Mud content (%)	W/C	SP (%)	Original diameter (mm)	Final diameter (mm)	Flow (%)
**LM00_1**	-	0	0.5	0	100	186	86
**LM30S150**	63–150	30	0.4	1	100	178	78
**LM35S150**	63–150	35	0.4	1	100	182	82
**LM40S150**	63–150	40	0.4	1	100	191	91
**LM20S63**	<63	20	0.5	0	100	212	112
**LM20S150**	63–150	20	0.5	0	100	205	105
**LM20S300**	150–300	20	0.5	0	100	199	99

Among specimens LM20S63, LM20S150 and LM20S300 with different particle sizes, it can be recognized that the smaller the particle size, the bigger the flow. This phenomenon needs further clarification. It may be related to the micro properties of the LUSI mud.

## 3. Experimental Details

The fresh mud was obtained from five different locations on the mud volcano site in Sidoarjo, East Java, Indonesia, in February 2012. The mud was then molded to be bricks-like shape, with 20 × 50 × 100 mm dimension. It was then oven dried at 100 °C for 24 hours in the oven before sending it for sintering at 910 °C for 5 hours. Sintering was performed in a furnace of a local roof tile manufacturer to enable sintering of mud in big volume. The sintered mud bricks were then ball-milled to three different fineness, *i.e.*, <60μm, 63–150μm and 150–300μm. The chemical composition of mud, both before and after sintering, was characterized by using X-ray Fluorescence (XRF) equipment, while examination of its microstructure was carried out by using X-ray Diffraction analysis.

Portland Pozzolan Cement (PPC) produced by local manufacturer was employed in this study, as the ordinary type 1 Portland cement is no longer available in the market. Four mixes were prepared with different degree of cement replacement at 0, 30, 35 and 40%. Mix with no cement replacement served as the control mix. Sand to cementitious ratio by mass was capped constant at 2.5. The fineness modulus (FM) of sand was 2.159.

The workability of the fresh mortar was measured by its flow diameter using Flow Table apparatus. Mortar was then cast into mortar cubes with 50 × 50 × 50 mm dimensions. Curing of mortar cubes were performed by immersing the mortar cubes in tap water at about 30^o^C until one day before the day of testing for compressive strength. Each compressive strength data presented in various tables and figures in this paper is a mean value of testing results of three cubes. Compressive strength was measured at 7, 14, 28 and 56 days of age to evaluate its strength development, as well as its strength activity index (SAI). Pozzolanic activity of LUSI mud was evaluated based on the SAI [9]. All tests were performed in accordance to relevant ASTM Standards.

## 4. Conclusions

This study reveals that the sintered LUSI mud of the mud volcano in Sidoarjo, Indonesia, has promising potential to be used as a pozzolanic material. Manufacturing semi high volume, up to 40% by mass, pozzolanic mortar incorporating LUSI mud is a possibility.

The smaller the particles size of the LUSI mud, the higher the compressive strength of the mortar as well as the higher the strength activity index (SAI). This is an indication of an increase in its pozzolanic activity due to reduction in its particles size.

With the increase in the amount of LUSI mud used as partial replacement for cement up to 40%, the rate of strength development is also increasing. This exhibits that the rate of pozzolanic activity development of LUSI mud is relatively low at early age; however it becomes faster at later ages, at least up to 56 days. Manufacturing semi high volume LUSI mud mortar, up to at least 40% as cement replacement, is a possibility, especially with a smaller particle size of LUSI mud, less than 63 μm.

The use of a larger percentage of cement replacement by LUSI mud does not show any adverse effect on the water demand, as the flow of the fresh mortar increased with the increase of percentage of LUSI mud usage. However, a more in depth exploration of the micro-level properties of LUSI mud needs to be carried out to unfold the mechanical and physical properties of LUSI mud.
